# Potential role of LPAR5 gene in prognosis and immunity of thyroid papillary carcinoma and pan-cancer

**DOI:** 10.1038/s41598-023-32733-y

**Published:** 2023-04-10

**Authors:** Ben Zhang, Lixi Zhang, Peng Qi, Renzhu Pang, Ziming Wang, Xuyao Liu, Qi Shi, Qiang Zhang

**Affiliations:** Thyroid Surgery Department, General Surgery Center, First Hospital of Jilin University, Changchun, 130000 Jilin Province China

**Keywords:** Cancer, Computational biology and bioinformatics, Data acquisition, Data processing, Cancer

## Abstract

Papillary carcinomas account for the largest proportion of thyroid cancers, with papillary thyroid carcinoma (PTC) being prone to early lymph node metastasis. Some studies have confirmed that LPAR5 can promote the progression of PTC, but immune-related analyses of LPAR5 and PTC have not been widely discussed. This study aimed to determine the role of LPAR5 in PTC prognosis and immunity. We will further explore the role of LPAR5 in 33 different tumor types. Regarding PTC, we analyzed the effect of LPAR5 expression on overall survival (OS). Gene Ontology (GO) and Kyoto Encyclopedia of Genes and Genomes (KEGG) analyses were performed. Immune-related analyses of immune checkpoints (ICPs) and immune cell infiltration were also performed. For pan-cancer, R packages were used to analyze prognosis, tumor mutational burden (TMB), microsatellite instability (MSI), and immune cell infiltration. Analysis of tumor microenvironment (TME) and ICPs was performed using Sangerbox (http://vip.sangerbox.com/home.html). The TISIDB database (http://cis.hku.hk/TISIDB/index.php) was used to identify immune and molecular subtypes. LPAR5 expression is associated with PTC prognosis and immunity as well as various human tumors. LPAR5 may be a potential biomarker for multiple malignancies and may provide a new target for cancer immunotherapy.

## Introduction

Thyroid cancer (TC) is the most common malignant endocrine tumor, affecting thrice as many women as men. Its incidence has been steadily increasing in many countries and regions over the past few decades^1,2^. Papillary thyroid carcinoma (PTC) is the most common pathological type of thyroid cancer, accounting for > 80% of all thyroid cancers, with a 10-year survival rate of > 95%^3,4^. Some patients have lymph node metastases at the time of diagnosis^5^. Although most PTC have a relatively good prognosis, tumor recurrence and metastasis still affect subsequent treatment and survival^6^. A number of patients cannot receive surgical treatment for various reasons, which greatly affects their survival.

Lysophosphatidic acid (LPA) is a phospholipid with numerous biological functions^7^, and acts as an extracellular signaling molecule by binding or activating LPA receptors (LPARs)^8,9^. Signal transduction between LPA and LPARs plays an important role in cancer occurrence and the development of cancer^7,10^. In various cancers, LPAR5 overexpression is involved in the invasion and metastasis of tumor cells^11,12^, and LPA can inhibit the activation and function of T cells by binding to LPAR5^13^. Recently, a study showed that LPAR5 expression was positively associated with the risk of cervical lymph node metastasis and activation of the PI3K/AKT pathway in PTC patients^14,15^. Zhang et al. also revealed that LPAR5 promotes the proliferation and migration of non-small cell lung carcinoma (NSCLC) by activating MLLT11^16^. Although LPAR5 and its associated AKT signaling pathway have been found to play an important role in the progression of PTC, immune-related differences between LPAR5 and PTC have not been widely discussed.

In this study, we analyzed the relationship between the expression level of LPAR5 and the prognostic value of PTC using R packages as well as the analysis of immune checkpoints (ICPs) and immune cell infiltration. Simultaneously, we also analyzed the role of LPAR5 in prognosis and immunology in 33 different types of cancers (Additional file T1). This study aimed to better understand the role of LPAR5 in anti-tumor immunotherapy in human tumors and to provide a new strategy for anti-tumor therapy.

## Results

### Prognostic value of LPAR5 in PTC

**Figure 1:**
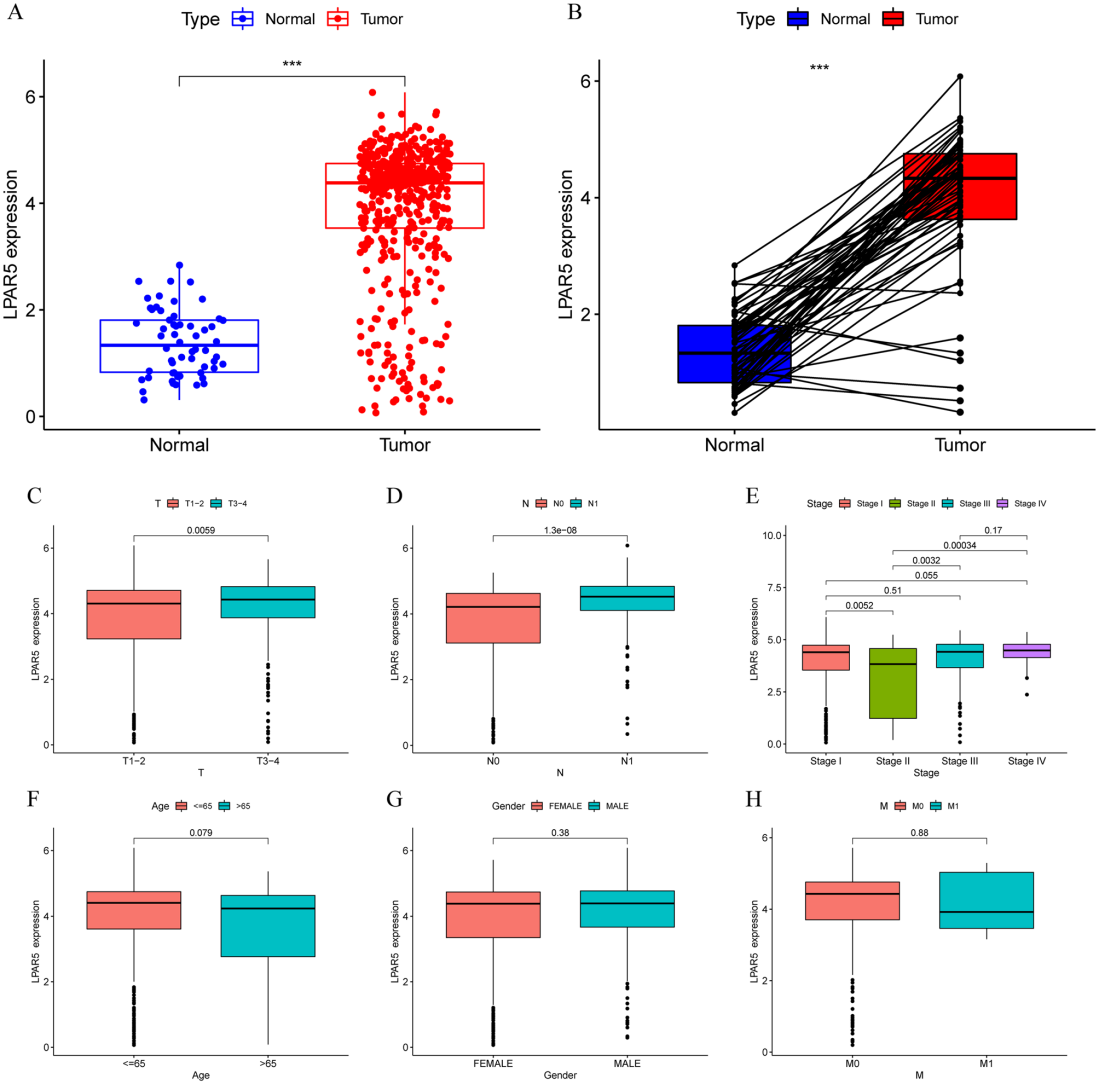
Expression of LPAR5 in normal thyroid tissues and tumor tissues and six clinicopathological features of patients with PTC. (**A**) LPAR5 expression was significantly different in 58 normal tissues and 510 tumor tissues. (**B**) LPAR5 expression between the paired tumor tissues and normal tissues in the same sample. (**C**–**E**) The expression level of LPAR5 was significantly different in T stage, N stage, and stages I–IV. (**F**–**H**) The expression level of LPAR5 was not significantly different in age, gender, and M stage. [Figures created by R, version 4.1.3].

As shown in Fig. 1A, there was a significant difference in LPAR5 expression between PTC tissue samples (n = 490) and normal thyroid tissue samples (n = 58) (p < 0.001), LPAR5 is highly expressed in tumor tissues. The same result was also obtained in the analysis of PTC samples and normal thyroid samples pairing difference (p < 0.001) (Fig. 1B). The expression level of LPAR5 was analyzed for each clinicopathological feature. The expression level of LPAR5 was significantly different in T stage, N stage, and stages I-IV of PTC patients (Fig. 1C–E). The expression of LPAR5 was lower in patients with T1-2, N0, and stage II disease. However, there were no significant differences in age, sex, and M stage (Fig. 1F–H).

**Figure 2:**
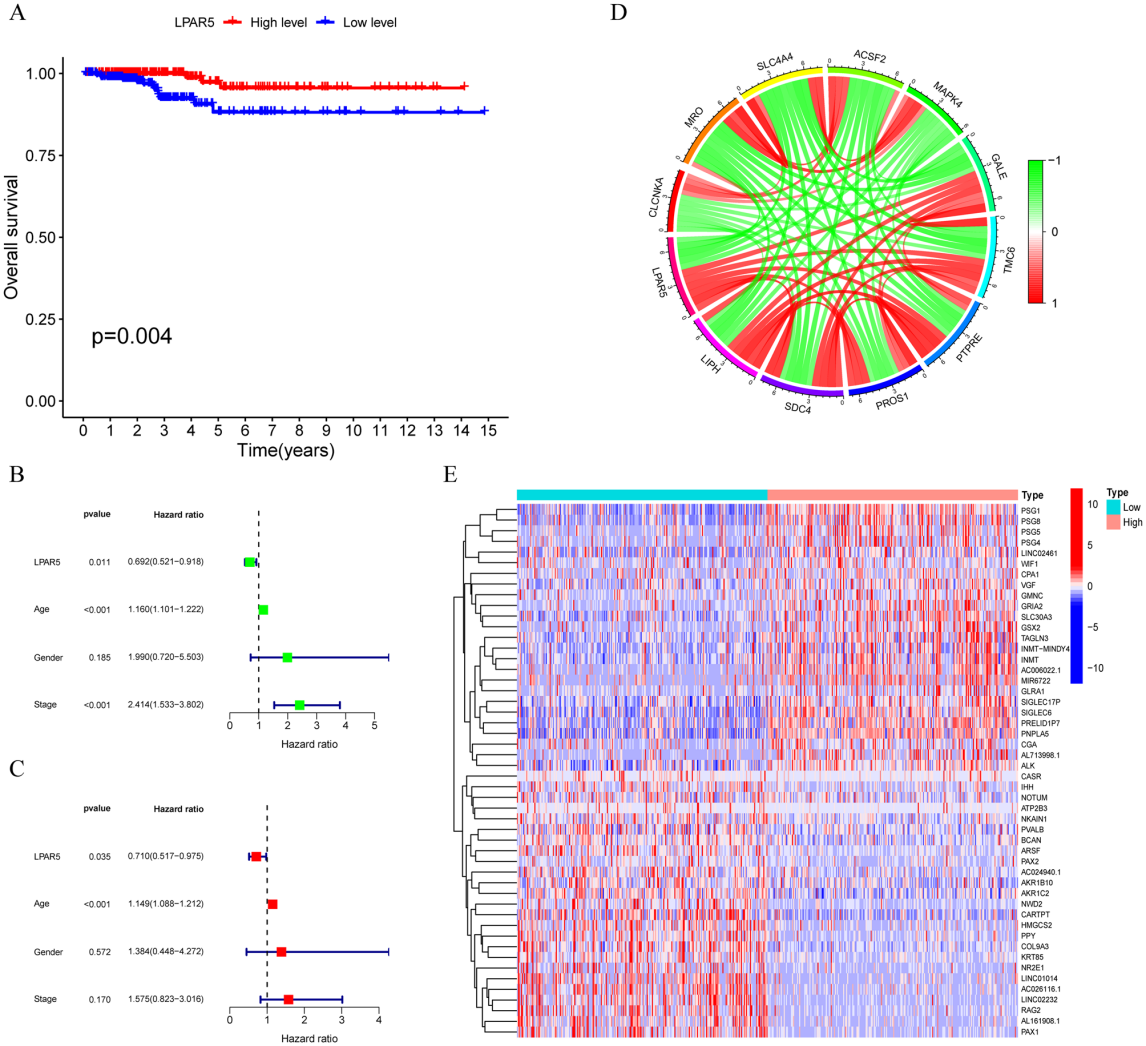
Survival analysis, univariate and multivariate Cox regression analyses, co-expressed analysis of LPAR5 in PTC, and expression of differentially expressed human genes in LPAR5 high and low expression samples. (**A**) LPAR5 is related to the survival time of patients, and with the increase of LPAR5 expression, the prognosis of PTC patients was better. (**B**, **C**) Uni-Cox and multi-Cox regression analyses showed that both age and expression of LPAR5 were independent risk factors for survival in PTC patients. (**D**) The six genes with the strongest positive correlation and the five genes with the strongest negative correlation with LPAR5. (**E**) The 25 most significantly up-regulated DEGs and 25 most significantly down-regulated DEGs in both high and low expression groups of LPAR5. [Figures created by R, version 4.1.3].

The Kaplan–Meier survival curve showed that high expression of LPAR5 was associated with a better prognosis than low expression of LPAR5 (p = 0.004) (Fig. 2A). Moreover, in uni-Cox and multi-Cox regression analyses, we concluded that the p-value of age and expression of LPAR5 was less than 0.05 (Fig. 2B,C), showing that both age and expression of LPAR5 were independent risk factors for survival in patients with PTC. Subsequently, the co-expressed genes of LPAR5 were analyzed, and a total of 65 genes with co-expression relationships to LPAR5 were obtained, of which 13 genes were negatively correlated and 52 genes were positively correlated. All results are shown in Additional file P1 (|cor| > 0.7, p-value < 0.001). We also visualized the six genes with the largest correlation coefficient and the five genes with the smallest correlation coefficient (Fig. 2D); the red and green lines represent positive and negative correlations, respectively. After analyzing the differences in genes between the two groups with high and low LPAR5 expression, we obtained 1566 differentially expressed genes (DEGs) (|Log_2_ FC| > 1, FDR < 0.05, and p-value < 0.05). A total of 299 genes were highly expressed in the LPAR5 high expression group and 1267 genes were highly expressed in the LPAR5 low expression group, and the top 25 DEGs in the two groups with high or low expression of LPAR5 were selected to create a heat map (Fig. 2E).

### Gene ontology, Kyoto encyclopedia of genes and genomes, and mutation frequency of tumor gene

**Figure 3:**
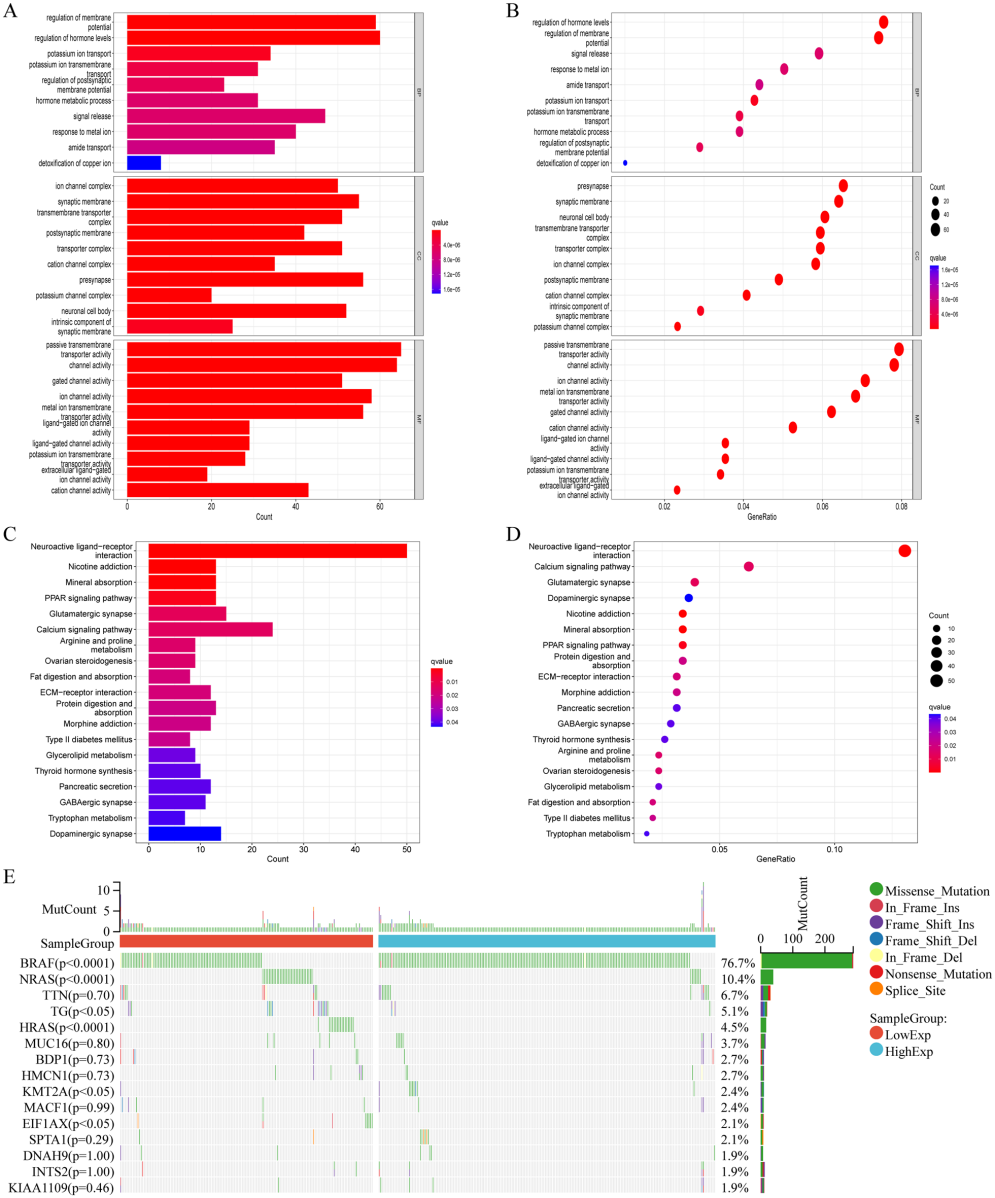
Gene ontology and Kyoto Encyclopedia of Genes and Genomes enrichment analysis and mutation frequency of tumor genes. (**A**, **B**) GO functional enrichment analysis shows that regulation of membrane potential, synaptic membrane, and passive transmembrane transporter activity are strongly associated with LPAR5 in the biological process, cell components, and molecular function. (**C**, **D**) Neuroactive ligand-receptor interaction is the most significant associated with LPAR5 in KEGG pathway enrichment analysis. (**E**) The mutation frequency of 6 tumor genes were significantly different between the high and low expression groups of LPAR5. [Figures created by R, version 4.1.3. Sangerbox website (version 3.0; http://vip.sangerbox.com/home.html)].

To explore the potential regulatory mechanisms of the 1566 DEGs, Gene Ontology (GO) and Kyoto Encyclopedia of Genes and Genomes (KEGG) enrichment analyses were performed. In the GO functional enrichment analysis, the ten GO projects with the most significant enrichment in the three parts are shown in Fig. 3A and B; these include regulation of hormone levels in biological processes, presynapse in cell components, and passive transmembrane transporter activity in molecular functions. Moreover, it was clear that LPAR5 was significantly enriched in molecular function. Similarly, 19 significantly enriched pathways were found in the KEGG pathway enrichment analysis, among which neuroactive ligand-receptor interactions were the most significant (Fig. 3C,D). Therefore, LPAR5 expression may regulate passive transmembrane transporter activity and multiple channel activity through neuroactive ligand-receptor interactions. There were significant differences in the mutation frequency of BRAF, NRAS, HRAS, TG, MKT2A, and EIF1AX between the high and low expression groups of LPAR5 (Fig. 3E).

### Immune-related analysis and drug prediction

**Figure 4:**
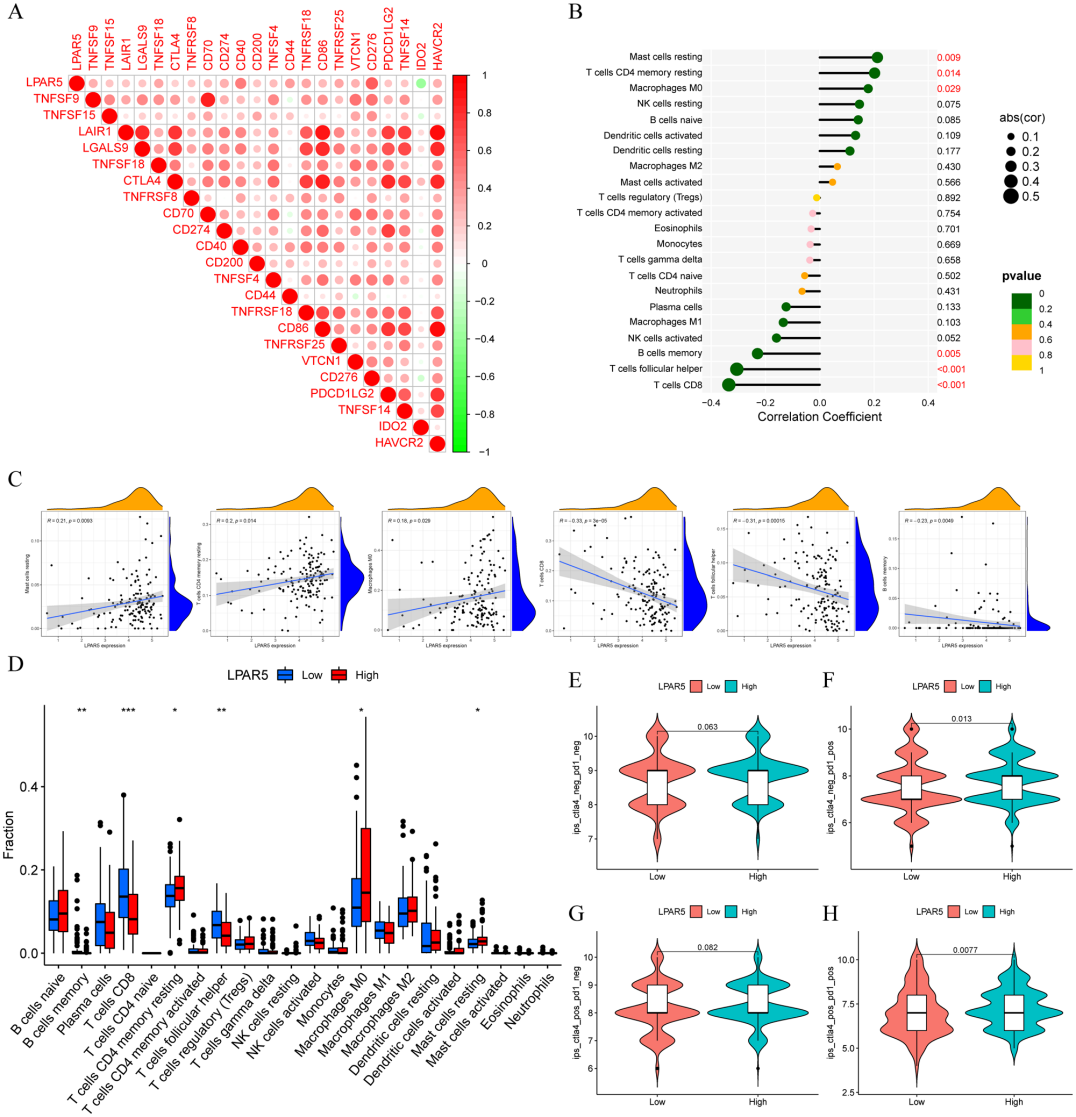
Analysis of immune checkpoints and immune cells and treatment response of PD1 and CTLA4. (**A**) 24 LPAR5-related ICP genes. (B and C) 6 LPAR5-related immune cells. (**D**) T cells follicular helper, T cells CD8, T cells CD4 memory resting, and Mast cells resting show differences in LPAR5 expression between the high and low expression groups. (**E**–**H**) Treatment response of PD1 monotherapy, CTLA4 monotherapy, and PD1 and CTLA4 combined therapy. [Figures created by R, version 4.1.3].

To study the immune function of LPAR5 in PTC, we conducted a series of immunoassays. First, we screened 24 LPAR5-related ICP genes (p < 0.001), and the gene with the largest correlation coefficient was CD276 (cor = 0.61). Among the 24 ICP genes, only one, IDO2, was negatively correlated with LPAR5 (Fig. 4A). We obtained six LPAR5-related immune cells (p < 0.05): mast cells resting, T cells CD4 memory resting, Macrophages M0, T cells CD8, T cells follicular helper, and B cells memory (Fig. 4B,C); the first three immune cells were positively regulated by LPAR5, while the last three were negatively regulated. In the follow-up difference analysis, we also found that the numbers of these six immune cells were significantly different between the two groups with high and low LPAR5 expression (Fig. 4D). In the immunotherapy analysis, it was found that PD1 monotherapy and PD1 combined with CTLA4 had a better effect in the LPAR5 high expression group, whereas CTLA4 monotherapy showed no significant difference between the two groups (Fig. 4E–H). Therefore, PD1 should be selected instead of CTLA4 for immunotherapy in patients with PTC with high LPAR5 expression. In the drug sensitivity analysis, we obtained 90 drugs with sensitivity differences between the high and low expression groups (p < 0.001); 57 drugs had lower IC50 values in the LPAR5 high expression group, and 33 drugs had lower IC50 values in the LPAR5 low expression group (Additional file P2A and B). The lower the IC50 value, the more sensitive the drug is to it.

### Comprehensive analysis of LPAR5 expression in 33 cancer types

**Figure 5:**
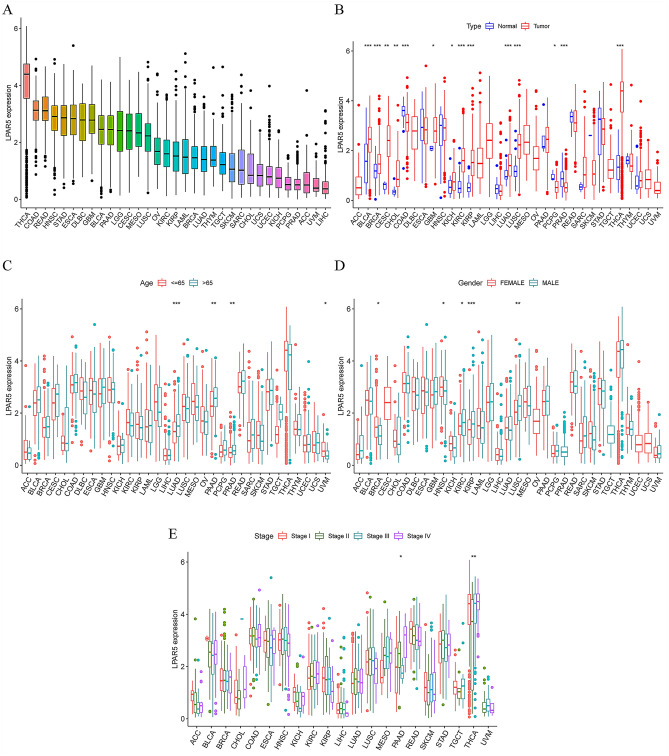
LPAR5 expression in 33 cancers. (**A**) LPAR5 expression levels in 33 different types of cancer. (**B**) LPAR5 expression levels in tumor samples and adjacent normal samples of 33 different types of cancer. (**C**) LPAR5 expression levels correlated with age in 4 of 33 different types of cancer. (**D**) LPAR5 expression levels correlated with gender in 5 of 33 different types of cancer. (**E**) LPAR5 expression levels correlated with stage in PAAD and THCA. [Figures created by R, version 4.1.3].

The expression levels of LPAR5 differed among the 33 cancer types, with the highest expression level in THCA and the lowest expression level in LIHC (Fig. 5A). The expression levels of LPAR5 were different between 14 types of tumor and normal samples (Fig. 5B). The expression levels of LPAR5 were higher in COAD, PCPG, and PRAD corresponding to normal samples, and the expression levels were higher in tumor samples of BLCA, BRCA, CESC, CHOL, GBM, KICH, KIRC, KIRP, LUAD, LUSC, and THCA. After analyzing the expression levels of LPAR5 in the three clinicopathological features, we can draw the following conclusions: in terms of age, LUAD, PAAD, PRAD, and UVM were significantly different among different age groups (Fig. 5C). In terms of gender, BRCA, HNSC, KIRC, KIRP, and LUSC showed significant differences between male and female groups (Fig. 5D). In terms of stage, PAAD and THCA showed significant differences from stage I to IV (Fig. 5E).

**Figure 6:**
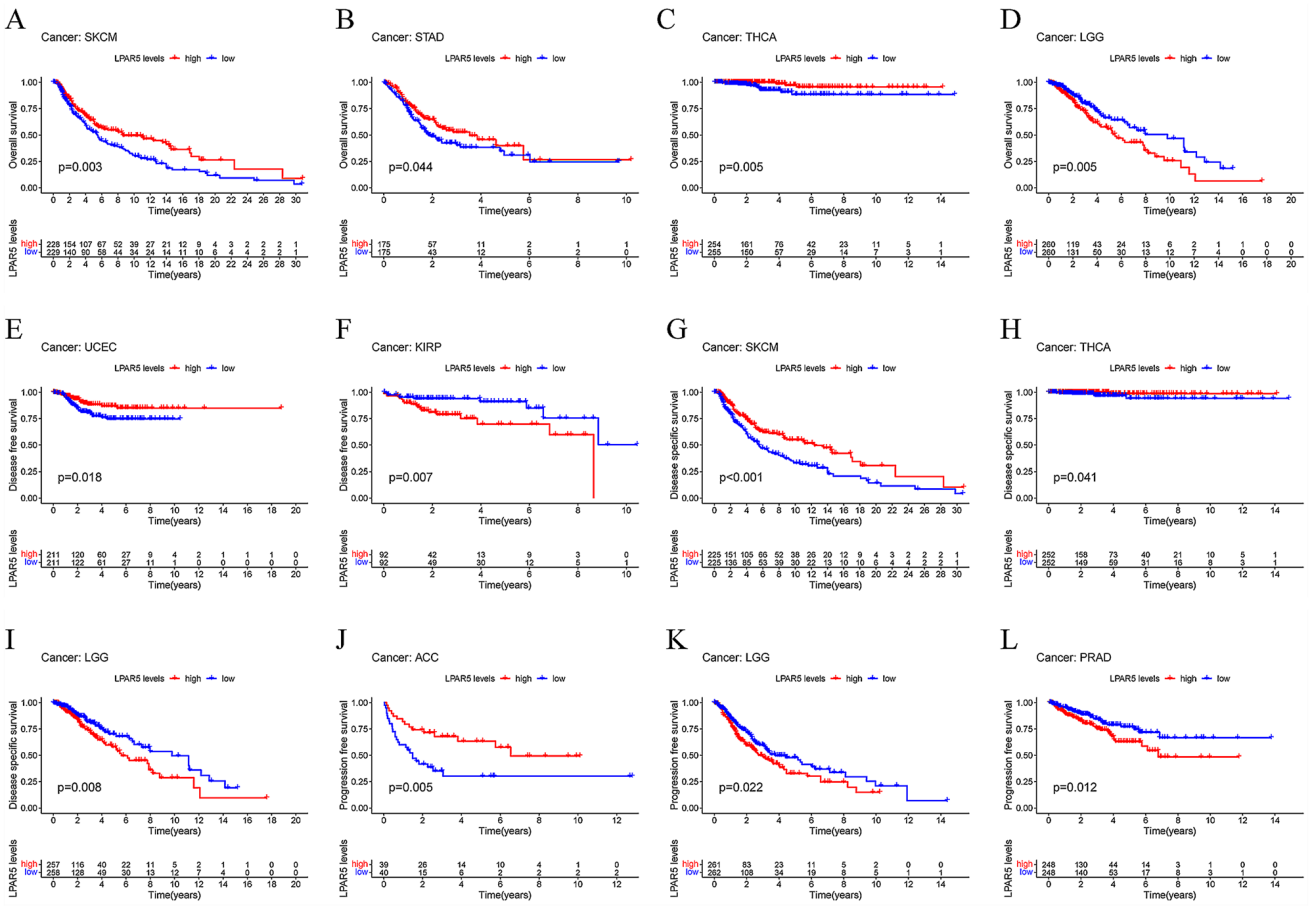
Survival analysis of 33 types of cancer. (**A**–**D**) The OS of SKCM, STAD, THCA, and LGG between high and low expression groups of LPAR5. (**E**, **F**) The DFS of UCEC and KIRP between high and low expression groups of LPAR5. (**G**–**I**) The DSS of SKCM, THCA, and LGG between high and low expression groups of LPAR5. (**J**–**L**) The PFS of ACC, LGG, and PRAD between high and low expression groups of LPAR5. [Figures created by R, version 4.1.3].

In the survival analysis of 33 cancer types, there were significant differences in the OS of the four cancers between the high and low expression groups. High LPAR5 expression was associated with better prognosis in SKCM, STAD, and THCA (Fig. 6A–C), but with poor prognosis in LGG (Fig. 6D). There were significant differences in DFS between the two cancers, among which high expression of LPAR5 was associated with better prognosis of UCEC, but with poor prognosis of KIRP (Fig. 6E,F). There were significant differences in the DSS of the three cancers, among which high expression of LPAR5 was associated with better prognosis in SKCM and THCA, but with poor prognosis in LGG (Fig. 6G–I). There were significant differences in PFS among the three cancers, among which high expression of LPAR5 was associated with better prognosis of ACC but with poor prognosis of LGG and PRAD (Fig. 6J–L).

GSEA was conducted according to the expression of LPAR5, and 8 and 15 cancers were significantly enriched in GO and KEGG, respectively. The five most significantly enriched functions or pathways are shown in the corresponding figures (Additional file P3A and B). The adaptive immune response was significantly enriched in the LGG, PRAD, SKCM, and UVM. The cytokine–cytokine receptor interaction pathway was significantly enriched in KIRP, LGG, LIHC, PCPG, PRAD, SKCM, THYM, UCEC, and UVM.

**Figure 7:**
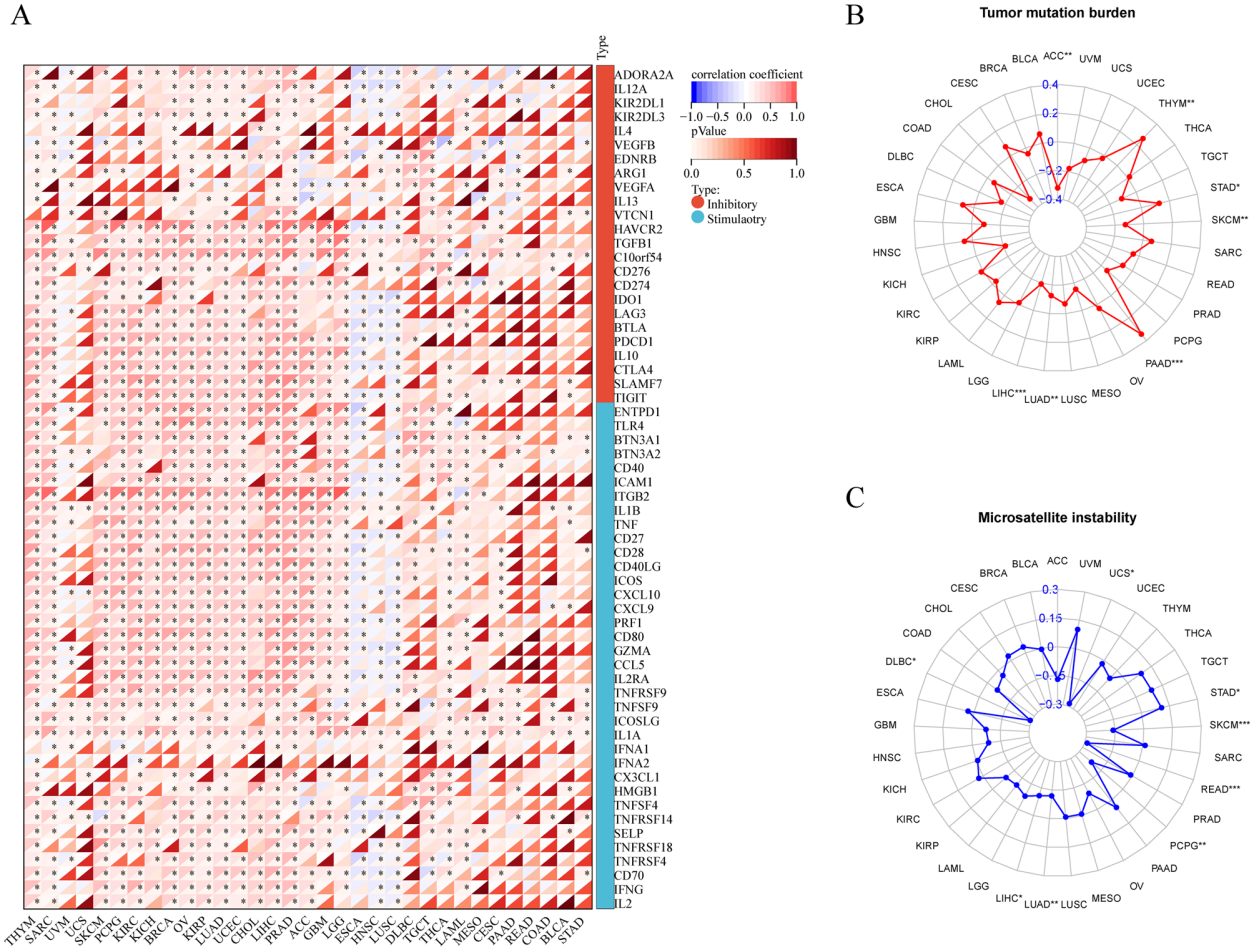
Analysis of immune checkpoint related genes, tumor mutational burden, and microsatellite instability. (**A**) Correlations between 60 ICP-related genes and the expression level of LPAR5 in 33 different types of cancer. (**B**) Association of LPAR5 expression level with tumor mutational burden for 33 different types of cancer. (**C**) Association of LPAR5 expression level with microsatellite instability for 33 different types of cancer. [Figures created by R, version 4.1.3. Sangerbox website (version 3.0; http://vip.sangerbox.com/home.html)].

Immune and stromal cells are the most important non-tumor components of the tumor microenvironment (TME), and there is evidence that their relative concentrations influence the efficacy of tumor-related immunotherapy. We used the Sangerbox online tool to calculate Spearman's correlation coefficient between LPAR5 and immune infiltrating score in each tumor to determine the immune infiltrating score with significant correlation. Finally, we observed that gene expression was significantly correlated with stromal cell infiltration in 10 cancer species: ACC, GBM, LGG, LIHC, PRAD, SARC, SKCM, TGCT, THYM, and UVM (Spearman's correlation coefficient > 0.5, and p-value < 0.001) (Additional file P4A), and gene expression was significantly correlated with immune cell infiltration in 13 cancer species: ACC, BRCA, GBM, KICH, KIRC, KIRP, LGG, LIHC, PCPG, PRAD, SARC, SKCM, and UVM (Spearman's correlation coefficient > 0.5, and p-value < 0.001) (Additional file P4B). Next, the correlation between LPAR5 expression levels and ICP-related gene expression levels was analyzed. The correlation between 60 ICP-related genes and the expression level of LPAR5 was calculated, and the expression level of LPAR5 was found significantly correlated with the expression level of multiple ICP-related genes in various cancer types (Fig. 7A).

The higher the TMB was, the larger the number of cancer mutations, and the mutations are recognized as neoantigens by the body. Therefore, a higher TMB would better activate T cells and have a better response to immune checkpoint inhibitors (ICIs) in clinic^17^. MSI plays an important role in the pathogenesis of cancer and is closely related to the occurrence, progression, and prognosis of many malignant tumors. Patients with microsatellite instability-high tumors (MSI-H) showed better results and prognosis after anti-tumor immunotherapy^18^. However, it is unclear whether the expression of LPAR5 is significant in TMB and MSI; therefore, we conducted relevant research. Spearman analysis was used to evaluate the correlation between LPAR5 expression levels and TMB according to the mutation data of the 33 cancers (Fig. 7B). We found that the expression level of LPAR5 was correlated with TMB in ACC, LIHC, LUAD, PAAD, SKCM, STAD, and THYM. We used the same method to analyze the correlation between the expression levels of LPAR5 and MSI (Fig. 7C), and the results showed that the expression level of LPAR5 was correlated with MSI in DLBC, LIHC, LUAD, PCPG, READ, SKCM, STAD, and UCS. In addition, analysis of immune cells (including B cells, T cells, NK cells, monocytes, macrophages, dendritic cells, mast cells, eosinophils, and neutrophils) showed that LPAR5 expression was correlated with immune cell infiltration in 20 out of 33 cancers (Additional file P5).

**Figure 8:**
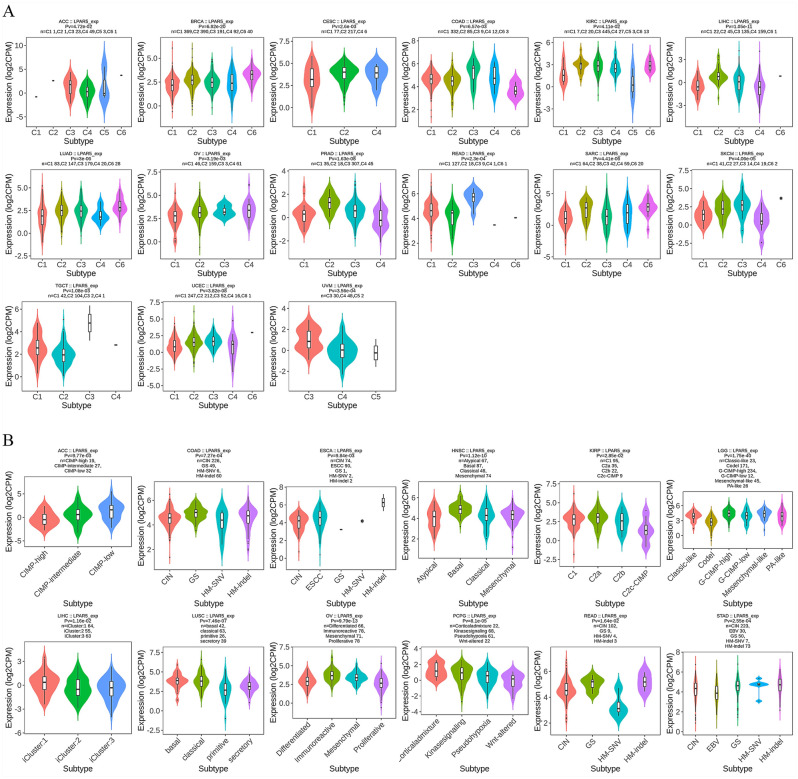
The correlation between LPAR5 expression levels and immune and molecular subtypes of various cancer types. (**A**) Immune subtypes. (**B**) Molecular subtypes. [Figures created by TISIDB database (http://cis.hku.hk/TISIDB/index.php)].

We then investigated the correlation between LPAR5 expression levels and the immune and molecular subtypes of the 33 cancers. The immune subtypes were divided into six categories: C1 (wound healing); C2 (IFN-gamma dominant); C3 (inflammatory); C4 (lymphocyte depleted); C5 (immunologically quiet); and C6 (TGF-b dominant). LPAR5 expression was associated with different immune types in the ACC, BRCA, CESC, COAD, KIRC, LIHC, LUAD, OV, PRAD, READ, SARC, SKCM, TGCT, UCEC, and UVM (Fig. 8A). Moreover, the expression level of LPAR5 varied among the different immune types in each cancer type. Using COAD as an example, LPAR5 was highly expressed in C3 and low in C6. Analysis of the molecular subtypes showed that LPAR5 expression levels were significantly correlated with different molecular subtypes of cancers in ACC, COAD, ESCA, HNSC, KIRP, LGG, LIHC, LUSC, OV, PCPG, READ, and STAD (Fig. 8B). The immune and molecular subtypes of the remaining cancers are presented in Additional File P6.

## Discussion

LPARs are involved in the regulation of lipid signal transduction^16^. Moreover, overexpression of LPAR5 promotes tumor progression through adsorption mechanisms^19^. Although LPAR5 has been identified as a potential marker of PTC, its immunological aspects have not been studied in PTC or many other cancers. Therefore, this study aimed to explore the role of LPAR5 expression in anti-tumor immunotherapy and its potential targets.

Our study validated LPAR5 as a prognostic value. The expression level of LPAR5 in PTC tissues was significantly higher than that in normal tissues. Based on survival and independent prognostic analyses of data downloaded from TCGA, age and abnormal expression of LPAR5 may be independent risk factors affecting the prognosis of patients, and LPAR5 expression may also be closely related to the occurrence of cervical lymph node metastasis. Therefore, it can be concluded that patients with low LPAR5 expression have a relatively poor prognosis, whereas high LPAR5 expression is closely related to cervical lymph node metastasis and higher T stage, which may indicate that the prognosis of PTC patients is not significantly related to the occurrence of cervical lymph node metastasis and tumor diameter. In co-expression analysis, the co-expression relationship between LIPH and LPAR5 was the most significant (R = 0.82, p < 2.2e−16). According to Gui et al., the expression of LIPH is significantly increased in PTC, and low LIPH levels can inhibit the progression of PTC^20^. High LIPH expression also promotes the metastasis of triple-negative breast cancer^21^. Zhuang et al. found that LIPH is a biomarker of poor prognosis in pancreatic cancer and its overexpression is closely associated with later tumor recurrence and staging^22^. Therefore, whether there is a certain connection between LPAR5 and LIPH through certain media is worth further exploration. MAPK4 is an atypical MAPK involved in the development of many human diseases^23,24^. Wang et al. demonstrated that MAPK4 promotes cancer progression by stimulating the AKT/mTOR signaling pathway, which is closely related to cell growth, survival, metastasis, metabolism, etc^25^. According to this study, MAPK4 and LPAR5 are negatively correlated in regulation, and the expression level of MAPK4 in the low-expression LPAR5 group was nine times higher than that in the high-expression LPAR5 group. Therefore, LPAR5 may indirectly affect PTC progression by affecting the AKT/mTOR signaling pathway.

GO analysis revealed that the DEGs between the two groups with high and low LPAR5 expression were significantly enriched in some functions regulating the transport of substances. These include the ion channel complex, passive transmembrane transporter activity, channel activity, ion channel activity, and gated channel activity. The most significant pathway of KEGG enrichment was the neuroactive ligand-receptor interaction. Neuroactive ligand-receptor interactions have been shown to be associated with various malignancies^26–29^. Studies have shown that stress is an adverse factor in tumor progression^30,31^. Stress has negative effects on both the neuroendocrine system and the sympathetic nervous system (SNS) of the body, which is manifested by continuous activation of the hypothalamic–pituitary–adrenal (HPA) axis and SNS, thus increasing catecholamine secretion, promoting tumor cell proliferation and metastasis, and enhancing angiogenesis^29,32,33^. Neuroactive ligand-receptor interactions are thought to be a key factor involved in emotion-related disorders^34–36^, and stress runs through these disorders. Wen et al. found that oxidative stress is involved in the pathogenesis process of PTC^37^. Therefore, the role of neuroactive ligand-receptor interactions in the occurrence and development of PTC deserves further exploration. There were five genetic mutations and LPAR5 expression exhibited significant correlation in BRAF gene mutation in patients. It is not difficult to find that the BRAF gene and the NRAS gene in the two groups were almost no simultaneous mutation. Previous studies have shown that simultaneous BRAF gene and NRAS gene mutations are virtually absent in individual colorectal cancer patients^38^. Whether this applies in PTC remains to be explored in future research.

In ICP analysis, we found that CD276 had the most positive correlation with LPAR5, which is involved in the regulation of malignant behavior of a variety of tumors and is also considered a potential target of immunotherapy^39,40^. CD276 plays a role not only in adaptive immune responses but also in innate immune responses^41^. CD276 is involved in the immune escape of squamous cell carcinoma stem cells^42^. Studies have confirmed that inhibition of CD276 can enhance the anti-tumor function of CD8^+^ T cells and NK cells and inhibit the growth of tumors^43,44^. Therefore, as a novel target for immunotherapy, CD276 should be paid more attention and studied. Whether CD276 is involved in the progression and regulation of the TME in PTC requires further confirmation. We also found that IDO2 was negatively correlated with LPAR5; therefore, the high expression of LPAR5 in PTC was accompanied by low expression of IDO2. As a rate-limiting enzyme in the kynurenine pathway, IDO2 participates in tryptamine metabolism, thereby affecting numerous physiological activities, including the immune response and neurotransmission^45^. The decreased expression of IDO2 can inhibit the migration and growth of tumor^46^, and IDO2 silencing in dendritic cells can promote the proliferation of T cells, which is expected to be a new target of immunotherapy^47,48^. Therefore, whether the improved prognosis of PTC patients with high expression of LPAR5 is related to the low expression of IDO2 and the interaction between LPAR5 and IDO2 warrants further study and discussion.

However, the role of LPAR5 in other cancers remains unclear. In this study, we explored the prognostic and immunotherapeutic value of LPAR5 in pan-cancer. The expression of LPAR5 varied between multiple tumors and corresponding normal tissues, and was higher in most tumor tissues. We also described the relationship between its expression differences and survival (OS, DFS, DSS, and PFS) and clinicopathological features (age, sex, stage, and TNM stages) in 33 cancers. The expression of LPAR5 was found to have the same function or enrichment of pathways in different types of cancer by GSEA analysis; for example, the adaptive immune response was enriched in LGG, PRAD, SKCM, and UVM. Immune response activation was enriched in PRAD, SKCM, and UVM. Cytokine-cytokine receptor interactions were enriched in KIRP, LGG, LIHC, PCPG, PRAD, SKCM, THYM, UCEC, and UVM. The chemokine signaling pathway was enriched in KIRP, LGG, LIHC, PCPG, PRAD, UCEC, and UVM. Cytokines and chemokines are essential components of the body's immune system, and the chronic inflammation they are involved in is an important marker of many types of cancer^49,50^. Therefore, we speculated that the expression of LPAR5 might be related to the immune function of cells.

Immune and stromal cells in the TME are typical representatives of non-tumor components, and their interactions affect gene expression, subsequent immunotherapy, and even patient prognosis^51,52^. According to the ESTIMATE algorithm, the stromal scores of ACC, GBM, LGG, LIHC, PRAD, SARC, SKCM, TGCT, THYM, and UVM and immune scores of ACC, BRCA, GBM, KICH, KIRC, KIRP, LGG, LIHC, PCPG, PRAD, SARC, SKCM, and UVM were all related to LPAR5 expression. The sum of the stromal and immune scores was called the ESTIMATEScore, and the higher the ESTIMATEScore, the lower the tumor purity. Furthermore, we used CIRBERSORT analysis to determine the relationship between the expression of LPAR5 and immune cell infiltration. By further sorting the results, it was found that macrophages were significantly correlated with infiltration in BRCA, HNSC, LAML, LGG, LUAD, OV, SKCM, TGCT, THYM, and UCEC; with NK cell infiltration in CHOL, KIRC, KIRP, LIHC, PRAD, THYM; and with CD8 + T cell infiltration in HNSC, KIRC, SARC, SKCM, THCA, UCEC, and UVM. CD8 + T cells are considered key factors in anti-tumor immunity^53,54^. KIRC had the most infiltrated immune cells (11 types). Although the relationship between LPAR5 expression and immune cells is not particularly clear, it may be involved in the formation of the TME in different types of cancer. In the TMB analysis, we observed the most significant correlation between the expression levels of LPAR5 and TMB in LIHC and PAAD (p < 0.001), suggesting that abnormal expression of LPAR5 might promote the occurrence and development of these two tumors by inducing gene mutations. We then explored differences in the immune and molecular subtypes of LPAR5 in various human cancers, and the results showed that there were indeed differences in various cancers, which also proved that LPAR5 may be a potential biomarker for pan-cancer.

The novelty of our study lies in the systematic and comprehensive evaluation of the value of LPAR5 expression in the expression, prognosis, immunity, and other aspects of 33 different types of cancer as well as the exploration of the functions and pathways that LPAR5 might affect or regulate. It would be helpful to understand the role of LPAR5 in tumorigenesis from the perspective of clinical tumor samples. However, this study had some limitations. First, we did not conduct in vivo/in vitro studies to verify the potential function of LPAR5. Second, although our results indicated a relationship between the expression of LPAR5 and the TME, TMB, and MSI, we did not know the mechanism involved. Third, LPAR5 as a new immunotherapy target still lacks direct evidence, and it will be necessary to verify whether LPAR5 can inhibit tumor growth or benefit the prognosis of patients through clinical trials of relevant targeted therapies. Therefore, the potential value of LPAR5 in tumor immunology needs to be further investigated in future studies.

## Methods

### Downloaded data from TCGA

TC transcriptome data (HTSeq-FPKM), including 510 thyroid cancer tissue samples and 58 normal thyroid tissue samples, and associated clinical data were downloaded from The Cancer Genome Atlas (TCGA, https://portal.gdc.cancer.gov). After sorting the samples, the samples containing both transcriptome data and clinical data were retained, and the samples with a survival time of less than 30 days were removed. In total, 490 PTC samples were obtained. Perl language (http://www.perl.org/) and the R software (version 4.1.3; https://www.r-project.org) were used for data analyses and collation.

### Prognostic analysis of LPAR5 in PTC

First, we used limma R package to verify the expression of LPAR5 between PTC and normal thyroid tissues, and PTC tissue was paired with normal thyroid tissue. Second, according to the median value of LPAR5 expression, patients with PTC were divided into two groups with high and low expression of LPAR5, referred to as the high and low expression groups, respectively. We analyzed whether there were differences in overall survival (OS) and LPAR5 expression in various clinicopathological features (including age, sex, stage, and TMN) between the two groups, and both were implemented using the survival R package and the limma R package respectively. We believe that a p-value < 0.05 represents a significant difference between the two groups. Third, univariate Cox (uni-Cox) and multivariate Cox (multi-Cox) regression analyses were used to assess whether LPAR5 and various clinical characteristics were independent risk factors for PTC, this step was done through the survival R package. Simultaneously, limma R package was used to screen the genes co-expressed with LPAR5 (Pearson correlation coefficients [cor] > 0.7, and p-value < 0.001) and compare the differential expression of human genes between the high and low expression groups of LPAR5 (|Log_2_ fold change [FC]| > 1, false discovery rate [FDR] < 0.05, and p-value < 0.05).

### Enrichment analysis and mutation landscape of gene expression

GO and KEGG analyses were performed using the clusterProfiler R package. GO analysis mainly included three aspects: biological processes, cell components, and molecular functions. KEGG analysis revealed a pathway enrichment^55–57^. The conditions for these studies were set as FDR < 0.05 and p-value < 0.05. The expression of LPAR5 and mutation frequency of tumor genes were analyzed using the Sangerbox website (http://vip.sangerbox.com/home.html).

### Immune-related analysis and drug sensitivity comparison

LPAR5-related ICPs were screened for PTC (p-value < 0.001). Among the 22 tumor-infiltrating immune cells (TIICs), we not only explored which levels of TIICs were correlated with LPAR5 expression (p < 0.05), but also analyzed the difference in TIICs between the high and low expression groups. Immunotherapy analysis was then performed between the high and low expression groups to compare the immunotherapy responses of the two groups to PD1 and CTLA4. This analysis was performed using the limma R package. We also assessed the treatment response based on the half-maximal inhibitory concentration (IC50) of PTC patients using the pRRophetic R package (p < 0.001).

### Comprehensive analysis

TCGA datasets for 33 cancer tissue samples and corresponding normal tissue samples used for pan-cancer analysis of LPAR5 were downloaded from UCSC Xena (http://xena.ucsc.edu/), including transcriptome, survival, and mutation data. These data can be used to analyze the differences in the expression levels of LPAR5 in normal and tumor samples, as well as in the clinicopathological characteristics (including age, sex, and stage) of 33 cancers. The OS, disease-specific survival (DSS), disease-free survival (DFS), and progression-free survival (PFS) were used to evaluate the prognostic value of LPAR5 in 33 cancers. Moreover, these 33 cancers were also analyzed using Gene Set Enrichment Analysis (GSEA), including GO and KEGG analyses (FDR < 0.25, and p-value < 0.05). All the above analyses were performed using the corresponding R packages.

Analysis of the TME and ICPs was performed using the Sangerbox website (http://vip.sangerbox.com/home.html). The data in Sangerbox is a unified and standardized pan-cancer data set downloaded from UCSC database, from which the expression data of LPAR5 gene and 60 marker genes of two types of ICP pathway [Inhibitory (24) and Stimulatory (36)] in each sample is extracted, and the samples with expression level of 0 are filtered out. Finally, stromal and immune scores of each patient in each tumor and the Spearman correlation between LPAR5 and marker genes of ICP pathway were calculated according to gene expression. Tumor mutational burden (TMB), microsatellite instability (MSI), and immune cell analyses were performed using the corresponding R packages. We then applied the TISIDB database (http://cis.hku.hk/TISIDB/index.php) to identify the immune and molecular subtypes^58^.

### Statistical analysis

The Wilcoxon test and t-test were used for LPAR5 differential expression analysis. Kaplan–Meier methods were used for survival analysis, and Pearson and Spearman’s analyses were used to study correlations. Cox regression analysis was used to explore the independent risk factors for PTC. Except for special instructions, P < 0.05 demonstrated that the difference was statistically significant.

### Statement

All methods in this study were performed in accordance with the relevant guidelines and regulations.

## Conclusion

In PTC patients, the expression of LPAR5 affects prognosis and is significantly correlated with many ICP-related genes and immune cells. These results of comprehensive analysis suggest that LPAR5 may be a potential biomarker for multiple malignancies and may provide a new target for cancer immunotherapy in the future.

## Supplementary Information

Supplementary Information.

## Data Availability

The databases used in this study are all publicly available and can be found in the TCGA database (https://portal.gdc.cancer.gov/), the UCSC Xena (http://xena.ucsc.edu/) database, the Sangerbox website (http://vip.sangerbox.com/home.html), and the TISIDB database (http://cis.hku.hk/TISIDB/index.php).
