# Supplementary material for: Potential role of LPAR5 gene in prognosis and immunity of thyroid papillary carcinoma and pan-cancer
Source: Sci Rep. 2023 Apr 10;13:5850. doi: 10.1038/s41598-023-32733-y (PMC10086052; doi:10.1038/s41598-023-32733-y)
Supplement: Supplementary file 1 — Supplementary Information. [file 41598_2023_32733_MOESM1_ESM.docx]

| **Abbreviation** | **Full** **Name** | **Abbreviation** | **Full** **Name** |
| --- | --- | --- | --- |
| ACC | Adrenocortical carcinoma | LUSC | Lung squamous cell carcinoma |
| BLCA | Bladder Urothelial Carcinoma | MESO | Mesothelioma |
| BRCA | Breast invasive carcinoma | OV | Ovarian serous cystadenocarcinoma |
| CESC | Cervical squamous cell carcinoma and endocervical adenocarcinoma | PAAD | Pancreatic adenocarcinoma |
| CHOL | Cholangiocarcinoma | PCPG | Pheochromocytoma and Paraganglioma |
| COAD | Colon adenocarcinoma | PRAD | Prostate adenocarcinoma |
| DLBC | Lymphoid Neoplasm Diffuse Large B-cell Lymphoma | READ | Rectum adenocarcinoma |
| ESCA | Esophageal carcinoma | SARC | Sarcoma |
| GBM | Glioblastoma multiforme | SKCM | Skin Cutaneous Melanoma |
| HNSC | Head and Neck squamous cell carcinoma | STAD | Stomach adenocarcinoma |
| KICH | Kidney Chromophobe | TGCT | Testicular Germ Cell Tumors |
| KIRC | Kidney renal clear cell carcinoma | THCA | Thyroid carcinoma |
| KIRP | Kidney renal papillary cell carcinoma | THYM | Thymoma |
| LAML | Acute Myeloid Leukemia | UCEC | Uterine Corpus Endometrial Carcinoma |
| LGG | Brain Lower Grade Glioma | UCS | Uterine Carcinosarcoma |
| LIHC | Liver hepatocellular carcinoma | UVM | Uveal Melanoma |
| LUAD | Lung adenocarcinoma |  |  |

Additional file T1: Abbreviation and full name of 33 different types of human tumors.


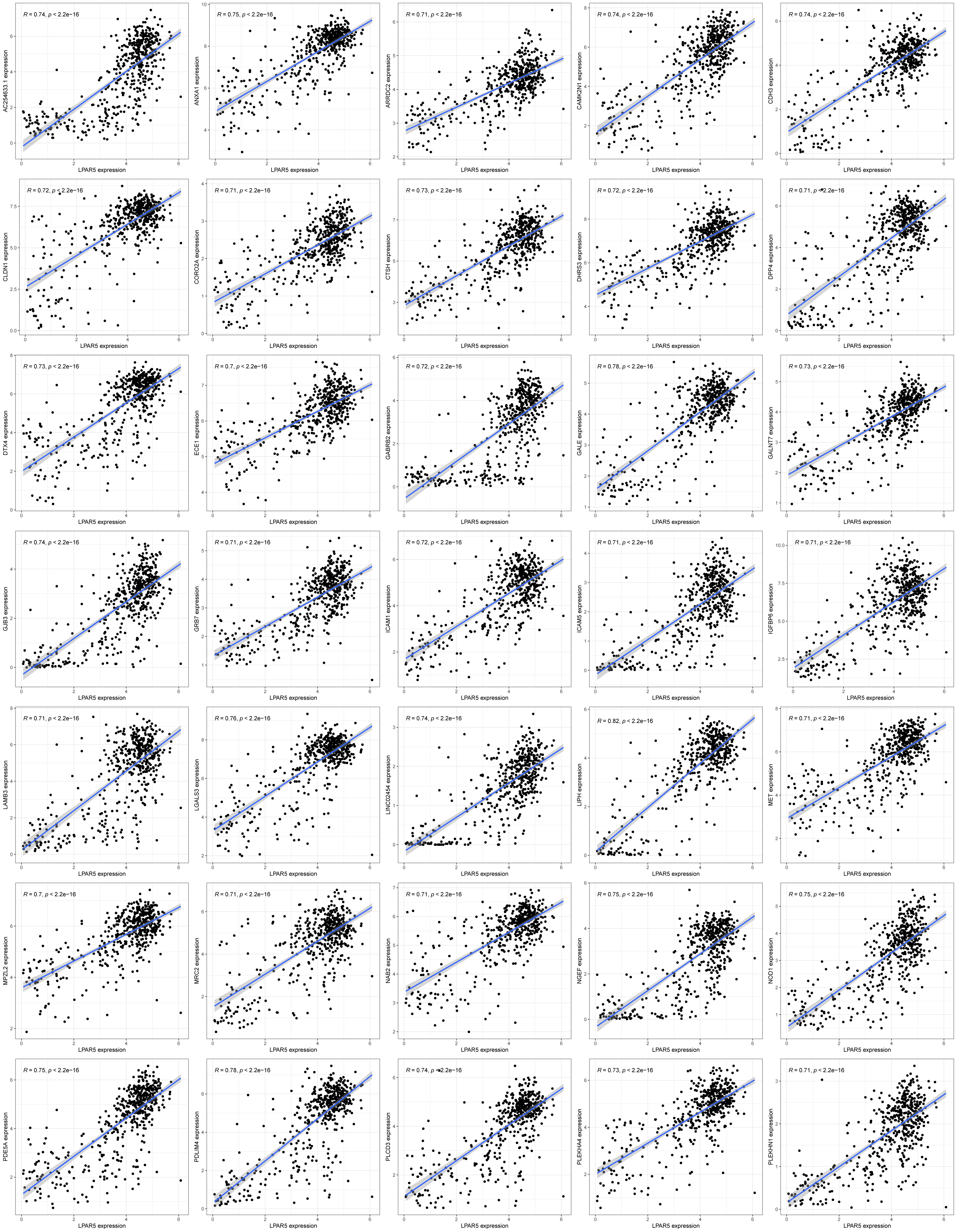


Additional file P1-1


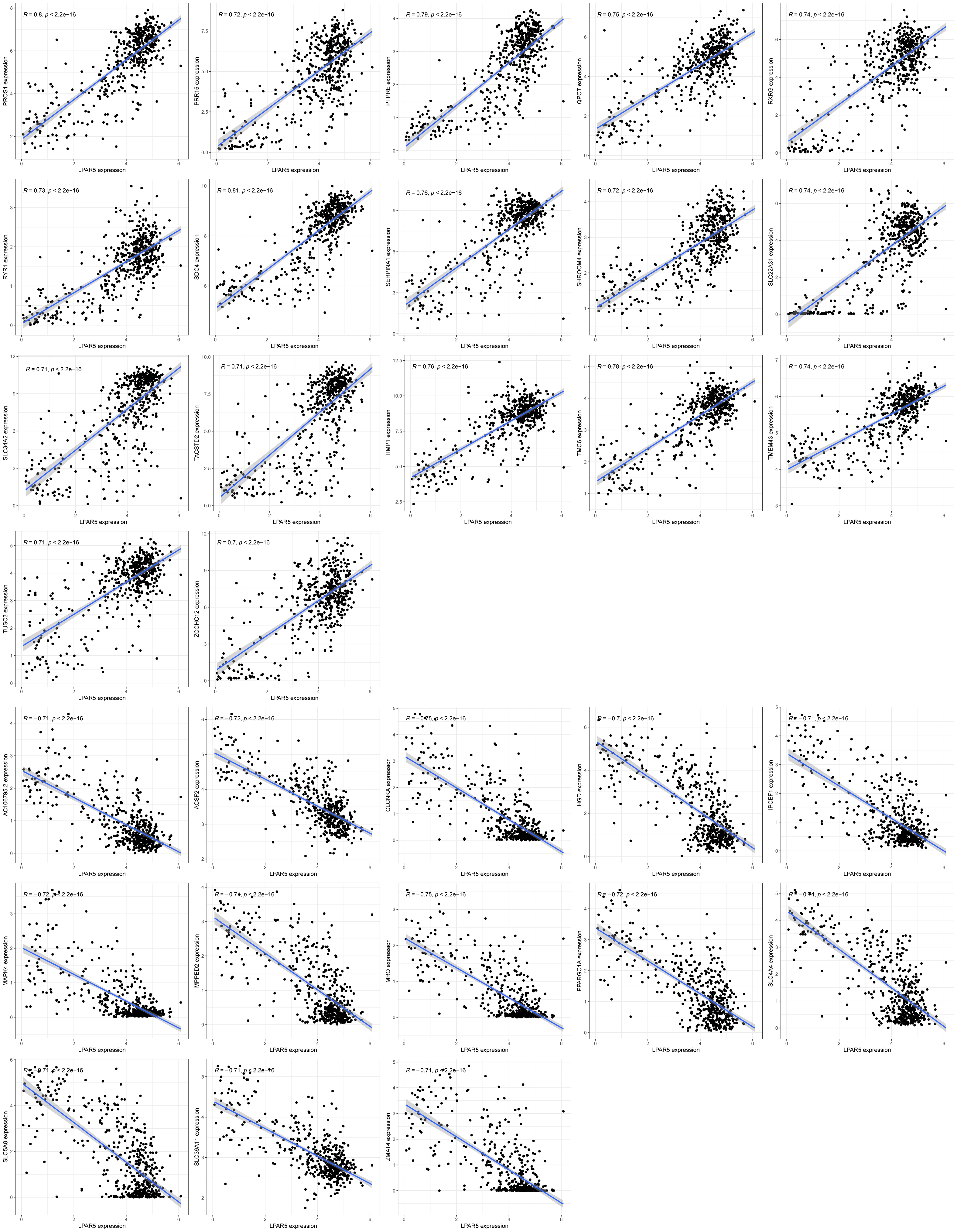


Additional file P1-2

Additional file P1: 65 genes with co-expression relationship to LPAR5. [Figures created by R, version 4.1.3.].


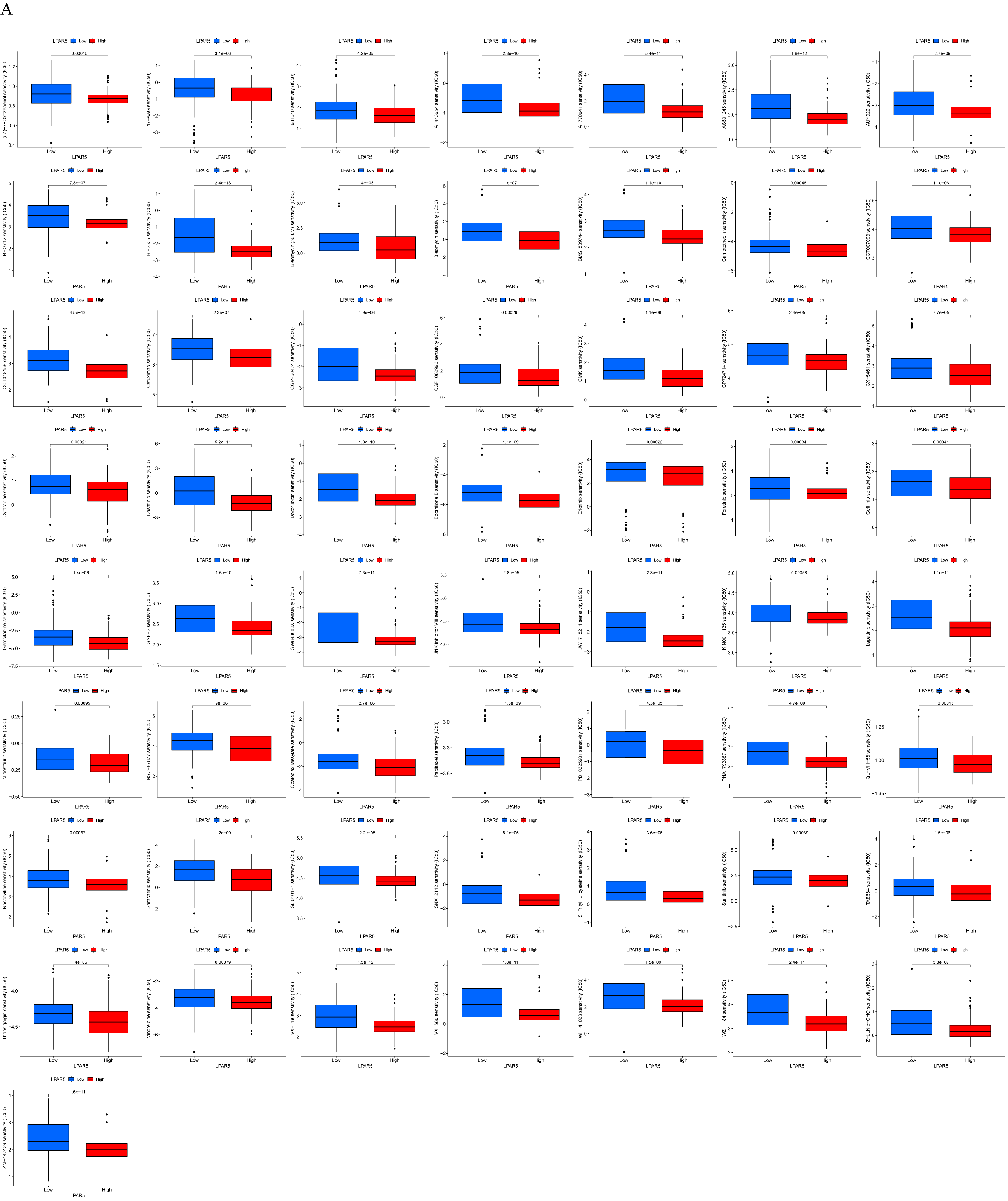


Additional file P2A


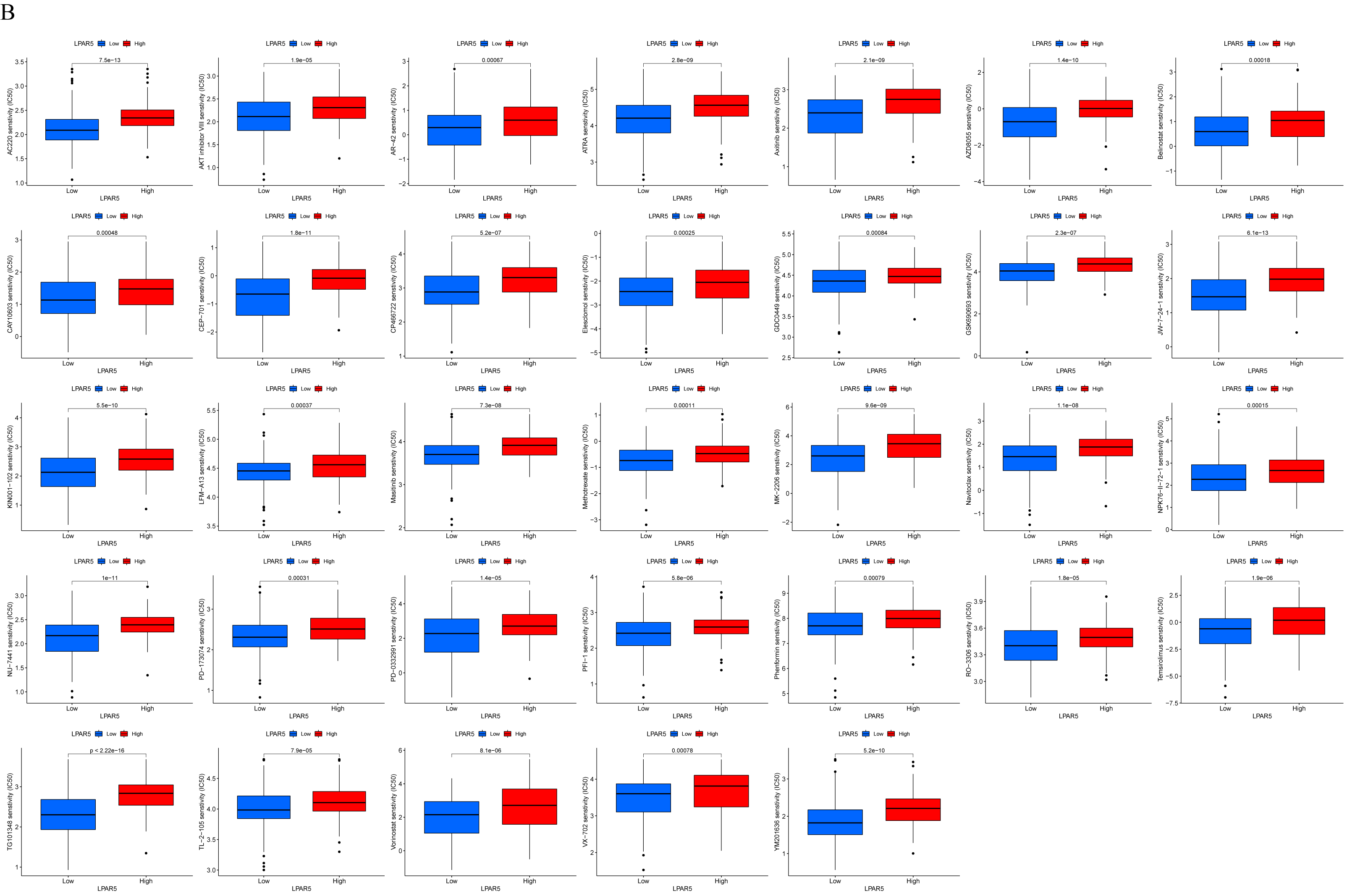


Additional file P2B

Additional file P2: Drug sensitivity analysis. (A) Lower IC50 values in the LPAR5 high expression group. (B) Lower IC50 values in the LPAR5 low expression group. [Figures created by R, version 4.1.3.].


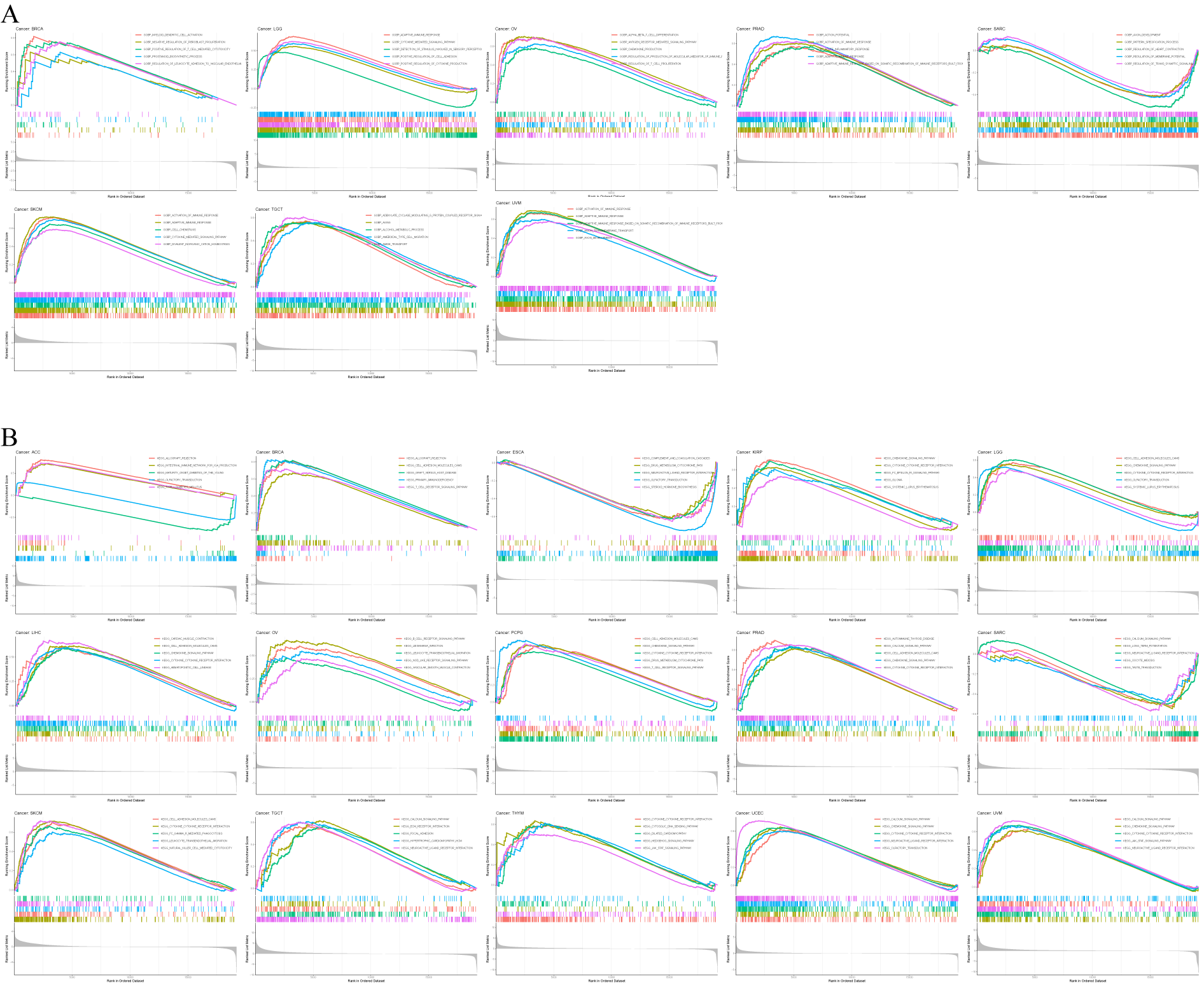


Additional file P3

Additional file P3: GSEA enrichment analysis. (A) GO enrichment analysis of BRCA, LGG, OV, PRAD, SARC, SKCM, TGCT, and UVM. (B) KEGG enrichment analysis of ACC, BRCA, ESCA, KIRP, LGG, IHC, OV, PCPG, PRAD, SARC, SKCM, TGCT, THYM, UCEC, and UVM. [Figures created by R, version 4.1.3.].


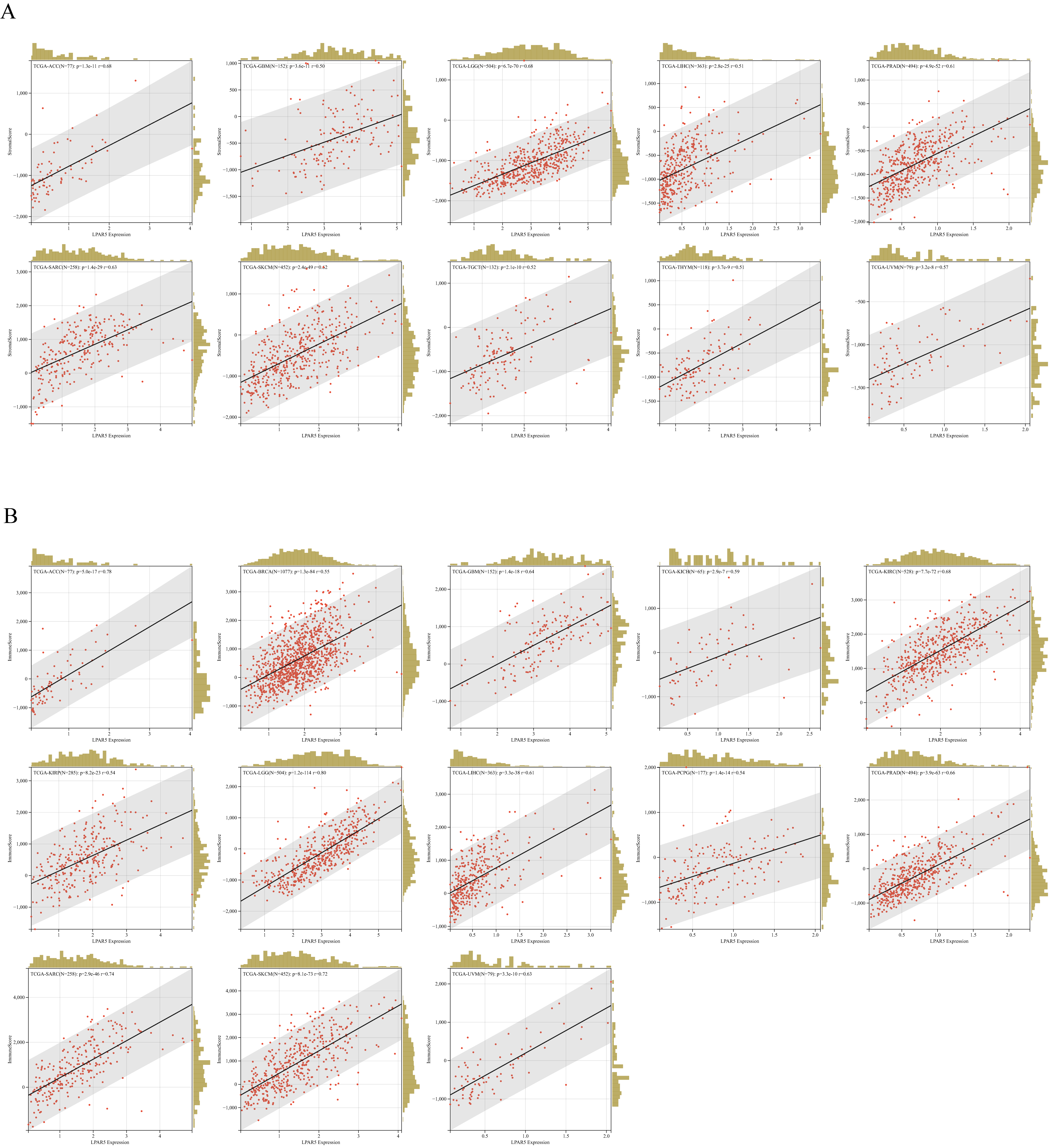


Additional file P4

Additional file P4: Analysis of tumor microenvironment. (A) Stromal cells infiltration in 10 cancer species. (B) Immune cells infiltration in 13 cancer species. [Figures created by Sangerbox website (version 3.0; http://vip.sangerbox.com/home.html)].


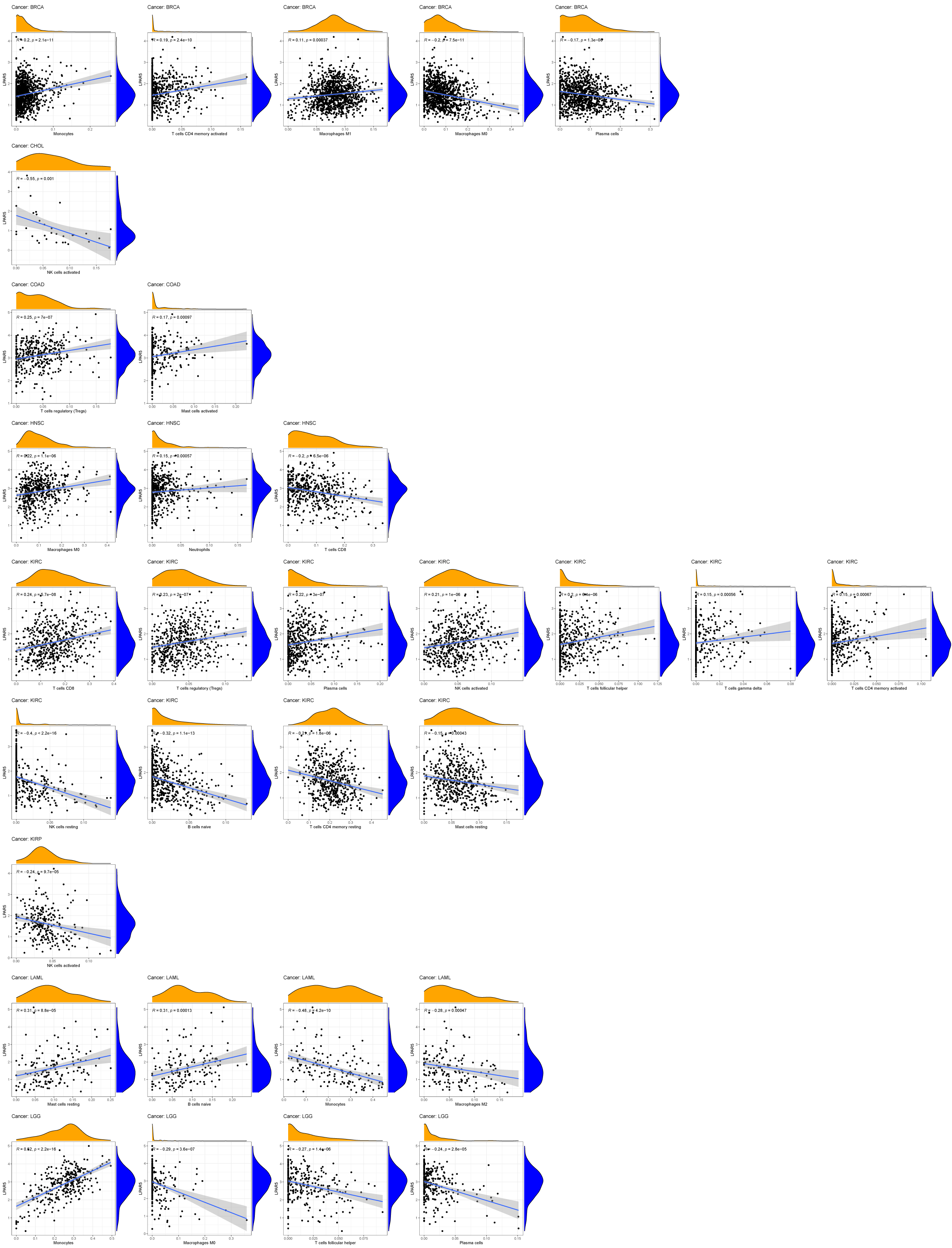


Additional file P5-1


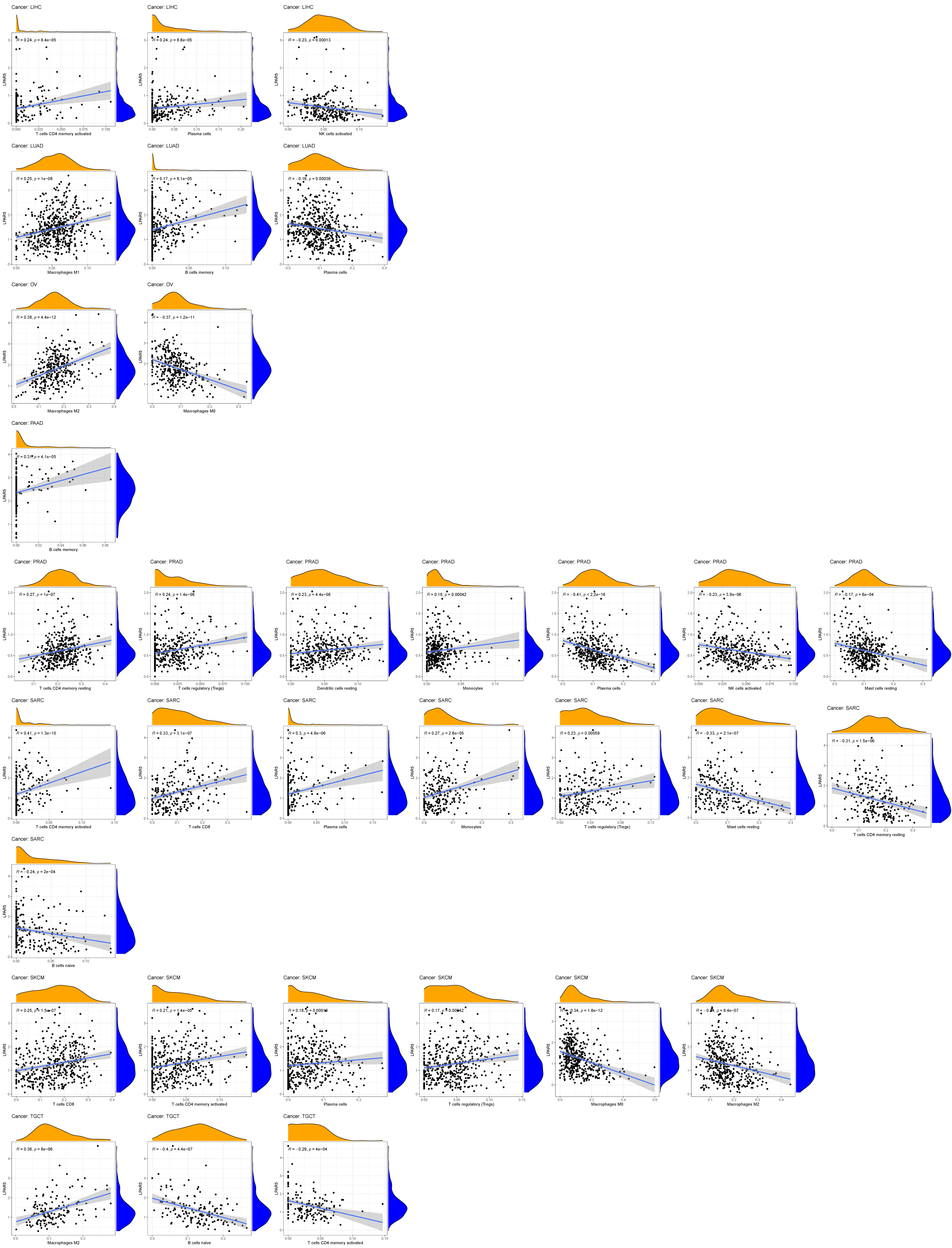


Additional file P5-2


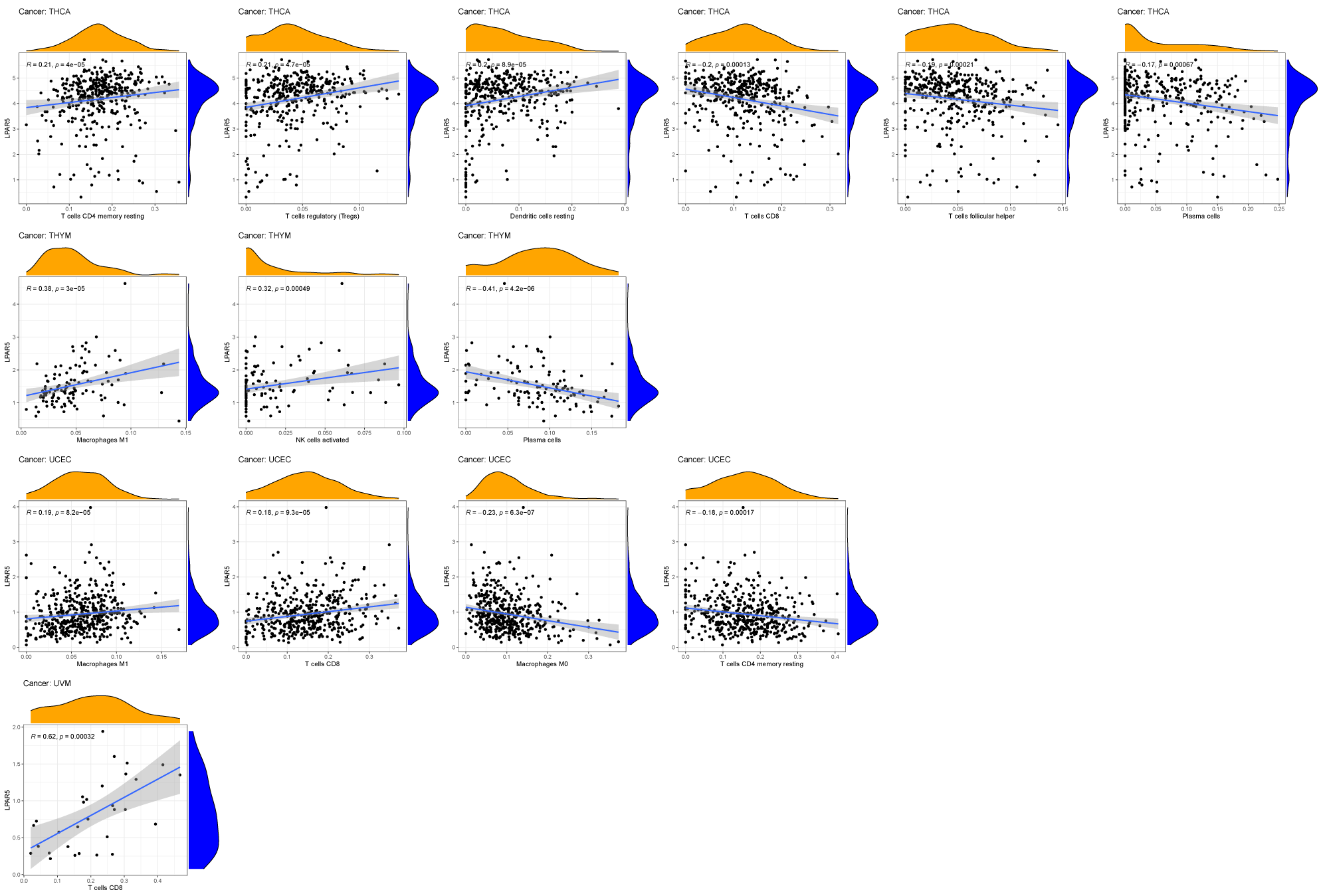


Additional file P5-3

Additional file P5: Relationship between LPAR5 expression and immune cell infiltration in 20 types of cancer. [Figures created by R, version 4.1.3.].


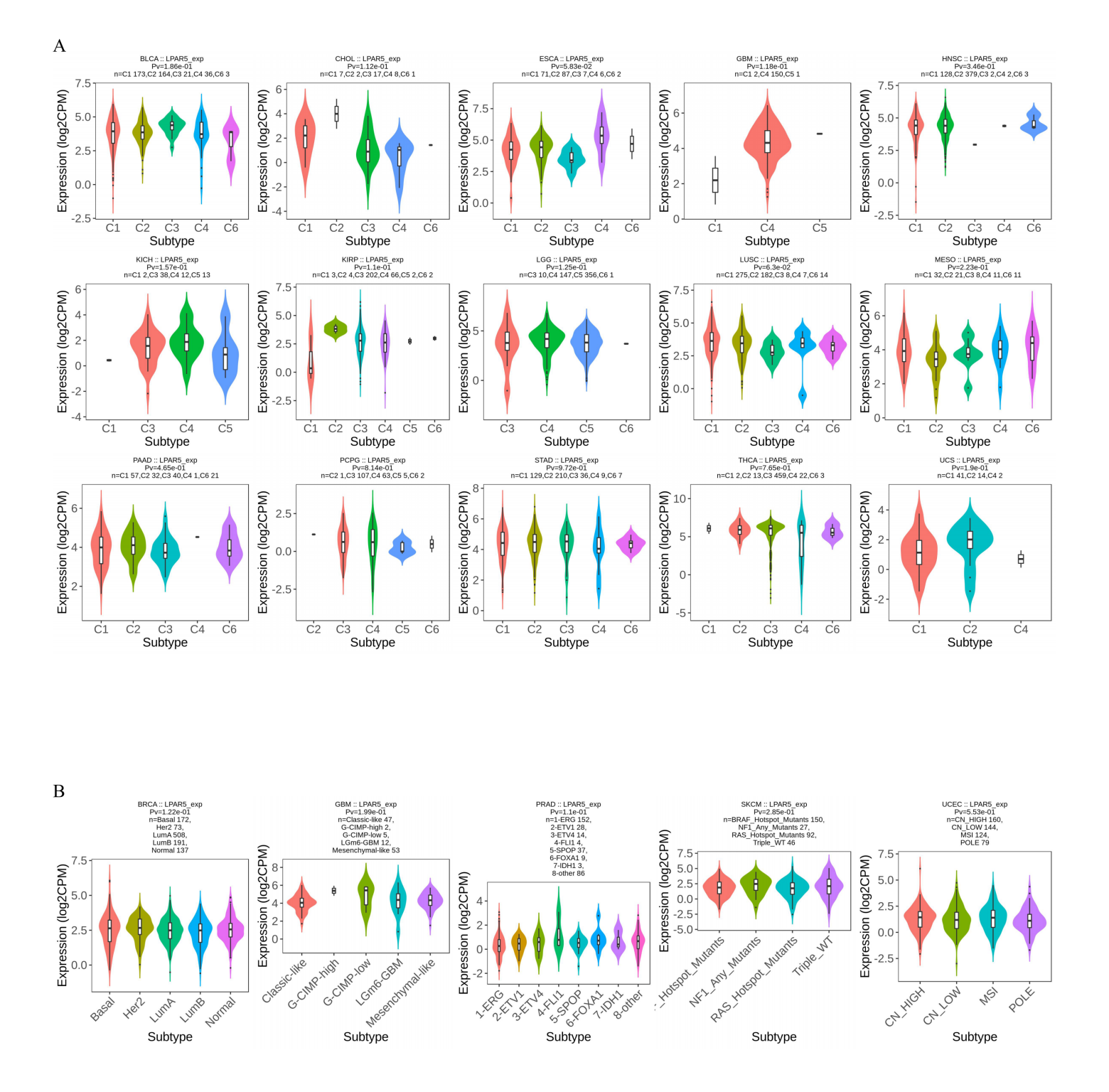


Additional file P6: The immune and molecular subtypes of the remaining cancers. (A) The immune subtype. (B) The molecular subtype. [Figures created by TISIDB database (http://cis.hku.hk/TISIDB/index.php)].
